# Genetic Variation and its Role in Malignancy

**Published:** 2011-09

**Authors:** Bente A. Talseth-Palmer, Rodney J. Scott

**Affiliations:** 1 *School of Biomedical Sciences and Pharmacy, University of Newcastle, Australia;*; 2 *Hunter Medical Research Institute, John Hunter Hospital, Newcastle, Australia;*; 3 *Hunter Area Pathology Service, Hunter New England Area Health, Newcastle, Australia*

**Keywords:** DNA damage and repair, environmental influence, genetic variation, malignancy

## Abstract

Genetic variation has long been thought associated with common complex disease and has therefore been widely studied. Genetic variation in the human genome is present in many forms and have been summarised in this review. The potential role of DNA damage, DNA repair and environmental influence on genetic variation in the development of cancer will be discussed, before significant genome projects are reviewed. All the various forms of genetic variation have been associated with malignancies and have been extensively studied and this is a review of the state of the field. We also discuss the road ahead in fulfilling the ultimate goal in all cancer genetic studies, which is decreasing deaths caused by cancer.

## BACKGROUND

Most diseases are multifactorial and are a result of an interaction between genetic and environmental factors that play a role in disease development throughout life. There is accumulating evidence indicating that genetic variation accounts for a proportion of susceptibility to common diseases such as diabetes, cardiovascular disease and cancer (1-3). The identification of genetic variation associated with common complex disorders is therefore a priority in elucidating the pathophysiological processes that underlie common human afflictions. In the past decades there has been an increasing interest in the possible impact of common, functional germline polymorphisms on clinical outcomes among patients with cancer.

Genetic variation refers to the genomic differences seen in a population or species (4). Because of the great diversity in the human genome, genetic variation is regarded as a parameter, which controls an individual’s phenotype (5).Genetic diversity refers to variation at the level of individual genes and provides a mechanism for population survival by their potential to adapt to an ever-changing environment. Within and between populations genetic variation has long been thought to be the key to the biology of human disease (6-8). Even though all humans are members of the same species no two individuals are exactly alike and even identical twins have slight differences in their DNA. Between any two humans, the amount of genetic variation is about 0.1 percent (9-11).

Genetic variation in the human genome is present in many forms and occurs at different frequencies throughout the genome. The different forms of genetic variation includes tandemly repeated DNA, single nucleotide polymorphisms (SNPs), small insertions/deletions, large-scale mutations, transposable elements, fragile sites and null alleles.

Tandemly repeated (satellite) DNA appears to be the main type of repeated DNA that accounts for the enormous variability observed in genome size among eukaryotes (12, 13). Tandemly repeated DNA can be divided into satellite DNA (highly repetitive DNA with repeat lengths of one thousand to several thousand base pairs (bp)), minisatellite DNA (moderately repetitive, 9-100 bp) and microsatellites (di-, tri- and tetra nucleotide repeats). Minisatellites are extremely polymorphic, which have made them excellent markers for DNA finger printing and linkage analysis (14-16). Microsatellites are very useful genetic markers (linkage analysis) as they are highly polymorphic, co-dominant and are spread approximately every 50 kb across the entire human genome (17, 18). Microsatellites therefore quickly replaced minisatellites and represented the markers of choice for gene mapping until SNPs were discovered.

SNPs represent the major source of genetic variation in the human genome and account for approximately 90 percent of all human genetic variation occurring approximately every 100 to 300 bases. For a variation to be considered a SNP, the least frequent allele has to have an abundance of 1 percent or greater in a given population (19, 20). According to the neutral theory of molecular evolution, most SNPs are maintained in natural populations due to their location in non-coding regions and as such their distribution is not under selective pressure (21). When occurring in a gene, SNPs can be a causative genetic variant that changes protein expression, directly contributing to a disease phenotype (22). Together with SNPs, small insertions and deletions account for the vast majority of genetic variation observed in DNA. Small insertions and deletions (1-30 bp) in the coding regions of genes can, but not always, lead to frame-shift mutations causing a severely altered and potentially non-functional protein.

Large-scale mutations in chromosomal structure include amplification and deletion of large chromosome regions known as copy number variation (CNV), translocations and loss of heterozygosity (LOH). From about the beginning of the last decade researchers highlighted DNA CNV as a large under-explored source of human genetic variation that may be a factor underlying a range of genetic diseases (23-26). Copy number refers to the amount of copies of a particular gene and CNV represents a copy number change involving a DNA fragment of 1 kilobase or larger and contributes to genomic diversity observed between humans (10). CNVs can include genes and their regulatory regions (27-29), suggesting that these variants may be important in our understanding of disease or phenotypic variation. The copy number of a particular gene can be related to gene expression levels and consequently the protein that it encodes (30).

Less frequent genetic variation includes transposable elements which can be divided into two main classes; DNA transposons and retrotransposons. DNA transposons in humans make up approximately 3 percent of the genome and appear to have become entirely inactive (31). Retrotransposons can be separated into long interspersed nuclear elements (LINEs) - AT rich region of 6-8 kb with internal promoters for RNA polymerase III, short interspersed nuclear elements (SINEs) - GC rich dimeric sequences of ~300 bp in size located in untranslated intronic regions and long terminal repeat (LTR)-retrotransposons. They make up about 21 percent, 13.5 percent and 8.5 percent of the genome, respectively (32, 33). Another form of genetic variation resides in rare fragile sites, which occur in less than 5 percent of the human genome and are comprised of di- and tri nucleotide repeats that may cause spontaneous breaks during DNA replication, frequently affecting neighbouring genes (34). Rare fragile sites are associated with the expansion of unstable repeat sequences (35, 36) and segregate in specific families. Common fragile sites in the human genome are part of the normal chromosomal structure; they are large regions of genomic instability and targets for chromosomal rearrangements and deletions (34). Null alleles, also a form of genetic variation, are the outcome of a mutant copy of a gene that lacks normal function due to the absence of a gene product, or the expression of a non-functional gene product (37).

DNA sequence variation can also be classified according to frequency. Mutations are rare sequence variants that are found in less than 1 percent of the population and typically have a major influence on disease development. Deleterious mutations can be subdivided into inborn error (embryonic lethal), germline mutations (hereditary disease) and somatic mutations, which are spontaneous mutations in cells that occurs throughout the body that could result in the malfunctioning of genes and consequently lead to common disease development (38). Polymorphisms are common DNA sequence variants that are classified as being present in over 1 percent of the population, and they are thought to have either a weak or no influence on disease development (39). However, the presence of multiple variations could potentially influence individual disease risk. The consequence of DNA sequence variation is genetic variability. Interpreting the functional significance of genetic variation in a population poses a major challenge since genetic variation between individuals is required for the long-term adaptation to environmental challenges (40).

Epigenetic variation, non-sequence based alterations (DNA methylation, histone modification and chromatin remodelling), that are inherited through cell division and variance in the reading frames of microRNAs (miRNAs) may also disrupt gene function (41-43). miRNA’s are small noncoding RNAs that regulate gene expression post-transcriptionally by binding of target mRNAs to regulate their stability or translation (44).

Individual susceptibility to cancer is influenced by the ability to repair DNA damage, sensitivity to DNA damaging agents and defects in DNA repair proteins, which have been associated with several human hereditary cancer syndromes (45-47). Genetic variation in DNA repair genes as a risk factor for disease is very apparent in rare cancers such as Ataxia telangiectasia, Fanconi’s anaemia, Nijmegan breakage syndrome, familial breast cancer, hereditary nonpolyposis colorectal cancer (HNPCC) and xeroderma pigmentosis (XP) (48-51). Genetic variation is now also being used as a tool to screen patients for targeted treatment. For example, the detection of somatically acquired mutations in the KRAS gene has been shown to interfere with the efficacy of new targeted treatments (EGFR-targeted mAbs) for bowel, head and neck, and lung cancers such that the treatment is only recommended to individuals harbouring the wild-type *KRAS* (52). The impact of genomic studies has been the ability to investigate the biology behind disease and cancer development in a comprehensive, unbiased, hypothesis-free manner (33, 53).

This review will discuss the potential role of DNA damage, DNA repair and environmental influence on genetic variation in the development of cancer, with a particular focus on the associations involving genetic variation and malignancy. As human genetic variation is an important topic in a rapidly changing genetics field this article is meant to be a review of the state of the field.

## DNA DAMAGE AND REPAIR

DNA is constantly exposed to external and internal mutagenic agents (i.e. free radicals, ionising agents, ultraviolet light, plant toxins and various chemical agents) that could potentially influence the integrity of the genome. If DNA damage is not repaired it can result in disruption of genomic integrity and alter the risk of malignancy. Many of the DNA lesions caused by mutagenic exposure are associated with structural damage, which can impact on the cells ability to function appropriately. DNA repair is not perfect and as such reflects a balance between maintaining genomic integrity and allowing sufficient differences to be transmitted from generation to generation thereby maintaining the potential of evolutionary change (54).

Failure to repair the effects of DNA damage can result in dysregulated cell growth which can ultimately result in cancer. It is therefore extremely important that the DNA repair system is constitutively active so it can respond to the induction of DNA damage. A wide range of intracellular products and bi-products can result in DNA damage which are considered to be major factors in mutagenesis, carcinogenesis and ageing (55, 56).

The DNA damage response is a series of molecular events that recognise, respond and result in DNA repair. The repair processes include removal of DNA damage, restoration of the integrity of the DNA helix, activation of DNA damage checkpoints, changes in the transcriptional profile that might be beneficial to cells and apoptosis (57, 58). Analysis of mutations in cancer cells has identified the importance of DNA damage response in preventing tumourigenesis (59, 60). The response to DNA damage implicates cell cycle checkpoint responses, preventing the expansion of cells that have sustained damage by initiating DNA repair or by activating the induction of apoptosis if there is too much damage (61).

In an individual cell, thousands of DNA lesions are repaired every day by different DNA repair systems that have overlapping repair specificities. Approximately 150 DNA repair gene products have been identified (62, 63) and there appears to be 6 major repair pathways: 1) DNA repair by direct reversal, 2) base excision repair, 3) mismatch repair, 4) nucleotide excision repair, 5) homologous recombination and 6) non-homologous end joining (58, 64-73). The six repair mechanisms function together to protect DNA from bi-products of cellular metabolism and environmental insult to maintain genome integrity.

The main function of chromatin is to package DNA into smaller volume and efficient DNA repair is complicated by the chromatin structure, a highly condensed structure that hinders DNA accessibility and its subsequent repair (64, 74). Despite this, DNA repair systems are extremely efficient. In normal cells, 50 percent of single- and double strand breaks induced by environmental ionising radiation are repaired within 15 minutes and complete repair occurs within 1-2 hours (75). Cancer patients have a significantly higher level of basal DNA damage than healthy controls (76). It has been shown that young female breast cancer patients have a much lower DNA repair capacity than their healthy female sibling, and that obesity might be a factor that is involved in DNA repair capacity (77).

Genetic variation, such as polymorphisms, present in DNA repair genes may have modifying effects on cancer risk influenced by the efficiency of DNA repair creating genomic instability. According to Madhusudan *et al*. (78) studies suggest that variation in DNA repair capacity in the normal population may influence cancer susceptibility and outcome, and deficiencies in DNA repair capacity have been associated with an increased risk of breast cancer (79). DNA repair polymorphisms have also been shown to be associated with individual levels of DNA damage, thereby modulating cancer risk (80). The modifying effects of genetic variation will only explain a small percentage of the differences in mutagen sensitivity observed in healthy individuals, which together with genetic variation in DNA repair genes, highlights the complexity of incorporating genetic differences into quantitative estimates of risk associated with environmentally relevant exposures (81).

## ENVIRONMENTAL INFLUENCE ON GENETIC VARIATION

The sources of exogenous damaging agents are numerous (i.e. ultraviolet light, x-rays, thermal disruption and man-made mutagens), causing genetic lesions (somatic mutations) as a result of insufficient DNA repair or excessive exposure. Repetitive elements (as described above) show a unique capacity to respond to environmental signals and tend to cluster in genes associated with externally triggered processes. Mutations in repetitive elements are associated with adaptive changes of phenotype in natural populations and certain types of mutations convey adaptive benefits (82). It is thought that environmental exposures early in development influences epigenetic changes, which may play a role in susceptibility to diseases later in life and disease phenotypes through modification of the epigenome (83, 84).

The development of cancer is an interplay between the accumulation of unrepaired DNA damage, epigenetic variation, environmental factors and DNA repair capacity, and an intricate balance between them is necessary to maintain genome integrity. For example, individuals exposed to high levels of benzene may have an increased risk for genotoxicity influencing cancer risk, due to decreased DNA repair capacity as a result of polymorphisms in susceptibility genes involved in xenobiotic clearance (85). Since both genetic and environmental factors influence the levels of enzymes that metabolically activate and detoxify chemicals (86), they are also considered to influence cancer risk. The relationship between genetic variation and environmental influence on disease risk has also been demonstrated between two non-synonymous *xeroderma pigmentosum D (XPD)* polymorphisms and sun exposure on skin cancer (87), and between a XPD polymorphism and the risk of breast cancer, especially from polycyclic aromatic hydrocarbon (PAH)-DNA adducts found in cigarette smoke (88). Furthermore, it has been shown that diets consisting of protective micronutrients as well as carcinogens and mutagens may alter the risk of malignancy, particularly in individuals who are genetically susceptible as a result of genetic variation (89). In addition, the intestinal microenvironment has been shown to influence tumour formation in an animal model of the inherited predisposition to colorectal cancer called familial adenomatous polyposis (90).

At a population level, confirmation of the interaction between environment and genetic susceptibility is observed in populations that have migrated from a region of low cancer incidence to one where the incidence is much greater. Neuhausen *et al*. (91) suggested that ethnic variation in cancer risk is most likely a result of both genetic and epidemiological factors based on the observation that Japanese men living in Japan was shown to have the lowest incidence of prostate cancer but with migration to the United States (US) the rate of prostate cancer increased.

## SIGNIFICANT GENOME PROJECTS

The Human Genome Project (HGP) began in 1990 with the intention of determining the DNA sequence and identifying all the genes in the human genome. The first part of the HGP was completed in 2003, which resulted in the identification of all the genes in the human genome (92). The study of genetic variation in other species increases the understanding of our own and consequently a series of genome have now been sequenced that include, bacteria (Escherichia Coli), the fruit fly (Drosophila melanogaster), rice (Oryza sativa), yeast (Saccharomyces cerevisiae) and the laboratory mouse (Mus musculus) (93-100) to name but a few. The first phase of the HGP has been completed and currently extensive re-sequencing is being undertaken, which is revealing considerable genetic variation both between and within species. The HGP has revealed that the spectrum of protein-coding genes is smaller than originally assumed and that differences between species are more likely to be a result of variation in the control of gene expression via regions of the genome that had hitherto not been assigned a function (101). The beneficial outcomes of the HGP and genome projects studying other species are many fold and include; earlier detection of genetic predispositions to disease, better understanding of the mechanisms modifying disease, energy and environmental applications (i.e. use microbial genomics to create new energy sources), risk assessment of disease, better understanding of human evolution and the common biology with other organisms, improved DNA forensics, and detailed knowledge of plant and other animal genomes, providing us the potential to develop stronger, more disease-resistant plants and animals (89, 93, 94, 102-106). The HGP genome technologies and bioinformatics tools have improved rapidly and the study of entire genomes, sets of expressed RNAs or proteins, gene families, variation among individuals, and the classes of gene regulatory elements are now being identified and their functional consequences assessed.

Only a fraction of the genome is comprised of genes encoding proteins, with the biological information contained in the genes nucleotide sequence. Coding regions in genes are thought to cover approximately 5 percent of the human genome. This is now being challenged by the results published by the ENCODE project (107), as the simple view of the genome as having a defined set of isolated loci transcribed independently does not seem to hold true. The human genome contains a considerable amount of information as it codes for not only the functioning of each and every one of us, but it is also a record for of an individual’s ancestry and origin. The ENCODE project has enriched the annotation of the human DNA sequence by describing the functional elements encoded therein (108, 109).

The international HapMap project developed a haplotype map of the human genome (110, 111), which describes the common patterns of genetic variation. The information from these projects has been made available to the public to increase the identification rate of genes associated with disease, individual drug responses and response to other environmental factors. Because of the great heterogeneity across the genome in terms of patterns of genetic variation, HapMap is one of the main online databases providing information on human genetic diversity (112).

## GENETIC VARIATION AND MALIGNANCIES

As early as 1974 it was suggested that cancers must exhibit a mutator phenotype (a series of mutations) as a result of differences between the paucity of spontaneous mutations and the large number of mutations found in human tumours (113). This observation has also been confirmed in more recent studies (reviewed by Prindle *et al*. (114)). Accumulation of mutations during neoplasia represents an imbalance between DNA damage, the efficiency of DNA damage repair and the response to un-repaired damage (115). Cancers arise as a result of an accumulative series of genetic and epigenetic changes that drive the progressive transformation of normal cells into highly malignant derivatives. An important factor for almost all cancer cells is genomic instability, ranging from the steady accumulation of mutations to gross chromosomal rearrangements and alterations in chromosome numbers (116-118).

Genetic variation does influencing the development of disease phenotype and cancer through different avenues, including genomic instability, chromatin structure and transcriptional activity (119-122). Most cancers are genetically unstable and most of the instability is observed at the chromosome level, with frequent gains and losses of large chromosome segments or entire chromosomes (123). A common pattern of unbalanced translocations, leading to loss of chromosomal material and gain of selected genes have been reported in the acute myeloid leukaemia complex karyotype (defined by the presence of abnormalities involving at least three chromosomes) (124). While losses and gains of chromosomal material have been observed in primary prostate tumours (125). Additionally, the Philadelphia chromosome, a specific chromosomal abnormality due to a reciprocal translocation involving chromosome 9 and 22, has been associated with chronic myelogenous leukaemia and is found in 95 percent of cases (126). This special translocation, also known as Bcr-Abl, has also been observed in acute lymphoblastic leukaemia and occasionally in acute myelogenous leukaemia (126).

The less frequently observed genetic variation (retrotransposons, null alleles and fragile sites) are also associated with different types of cancer. The distribution of retrotransposons has been implicated as a potential source of disease by insertional mutagenesis or their ability to influence transcription of neighbouring genes (32, 127). SINEs have been associated with different types of cancers; leukaemia, ovarian carcinoma and breast cancer (128-131). Null alleles of the gene glutathione-S-transferase (GST) M1 and/or T1 have been implicated as a risk factor for lung cancer, ovarian cancer, breast cancer, bladder cancer and cancer occurrence in hereditary non-polyposis colorectal cancer (HNPCC) (132-136). There have been studies showing that fragile sites, and associated genes, are frequently deleted or rearranged in cancer cells and this has demonstrated their importance in genomic instability in tumourigenesis (137-146). FRA2B (3p14.2) and FRA16D (16q23.2) are the two most frequently expressed common fragile sites in the human genome (147, 148). *FHIT* and *WWOX* (the genes located within FRA3B and FRA16D respectively) have both been shown to function as tumour suppressors genes, and their inactivation have been associated with a poor clinical prognosis in cancer (61, 149). Minisatellites have been associated with fragile sites and are proximal to a number of recurrent translocation breakpoints (150). A minisatellite downstream of the H-ras proto-oncogene has been associated with the risk of cancer (151). However, from 1985 and for the following 15 years, contradictory results were published on the association of this minisatellite near the H-ras gene and cancer risk. A more recent study using improved genotyping and analysis method has failed to reproduce the association (152) suggesting that variation at this site is more complex than originally thought.

Approximately 10 percent of all cancers are familial, which is defined as cancer that occurs within families at relatively high frequency and at a younger diagnostic age compared to the general population. Conversely, approximately 90 percent of cancer cases consist of non-familial, sporadic forms of cancer (153). Genetic predispositions to sporadic cancer are considered to be multifactorial, but a study on non-familial breast cancer indicated that predispositions to sporadic cancer are strongly influenced by genetic factors (154). Predisposition to disease is a combination of weak genetic variants that may be of much more significance to public health than the marked individual risk seen in the inherited cancer syndromes (155, 156). Nonsense mutations have frequently been identified in a number of inherited predispositions to cancer and include adenomatous polyposis (157), hereditary nonpolyposis colorectal cancer and sporadic colorectal cancer (158, 159), familial breast cancer (160) and multiple endocrine neoplasia type 1 (161). The identification of tumour susceptibility genes has significantly aided our understanding of the pathogenetic mechanisms underlying cancers that appear not to be associated with an inherited predisposition (162).

Over the past decade there has been an increasing interest in the possible impact of common, functional germline polymorphisms on clinical outcomes among patients with cancer (163-171). For example, a Caspase-8 (CASP8) polymorphism has been associated with reduced susceptibility to multiple cancers (172) while low-penetrence CRC susceptibility loci have been shown to increase the risk of developing colorectal cancer in Lynch syndrome patients (173, 174). When searching for SNPs associated with disease it is important to consider that the frequency of the variation of interest can vary significantly between populations. The allele frequency for any given SNP tends to be population-specific (175-177), but it has also been shown that for most of the common disease associated SNPs, ethnicity is likely to be a poor predictor of an individual’s genotype (178).

Identifying genes that contribute to complex disease has been and remains a major challenge. Substantial scientific debate has been generated regarding optimal strategies to localise and identify genes for complex human disorders (9, 179-184). Linkage analysis has been used successfully to map highly penetrant genes associated with monogenic disease (185), but has been less successful for the identification of low-penetrant susceptibility genes. Association studies utilising candidate gene polymorphisms that are likely to affect the tumour development were excellent for the purpose of identifying common genetic variation that confers modest disease risk. Both these approaches have now however been superseded by a genome wide approach (186-189). Both large population based studies, comparing thousand of subjects with equal number of controls, and inherited predisposition disorders are being utilised to examine genetic variation, and its association with disease (190-192).

Genome-wide association studies (GWAS’s) based on the common disease - common variant hypothesis (193) have appeared as a relatively new approach for investigating the genetic basis of complex disease (194). A GWAS is designed to examine the entire genome using a large number of markers (some of which are linked to a disease allele) to discover gene loci that are different between individuals with disease compared to those without disease (195). These studies require thousands of samples (both cases and controls) to have sufficient power to detect susceptibility loci as they suffer from the problem of multiple testing that must be corrected for. GWAS’s has served as an attractive approach to search for novel moderate to high-penetrance genes in high risk cancer families, as both common and rare variation may cause cancer susceptibility. In 2008 it was suggested that the variation identified by GWAS’s only explain a small fraction of the overall disease risk in any given disease and from a population-wide perspective their impact seems limited (196, 197). By 2009, 600 human GWAS’s examining 150 diseases were reported that found 800 SNP associations (198). Many of these studies have investigated a variety of cancers types, which started to appear in leading journals around 2007 (190, 192, 199).GWAS’s have identified over 100 low-penetrant cancer susceptibility loci associated with modest disease risk (OR<1.5) (200, 201) and this has cast some doubts over the validity of the common disease - common variant hypothesis (193). Rather than identifying single gene associations it is more likely that GWAS results will reveal molecular pathways associated with disease. The importance of replication studies for GWAS has been emphasised as only a limited number of observed variants are true risk alleles (202). Even though GWAS’s have enhanced our ability to study genetic variation, the targets identified by these studies require validation through functional studies before the findings can be used in cancer prediction and prevention (200, 202). GWAS’s will, however, continue to reveal new insights into tumour biology (203). Nevertheless, to fully understand the genetic basis of common malignancies a more integrated approach that includes a combination of SNP, CNV and whole-genome sequencing data will be required to provide clinically relevant information.

CNVs were discovered only after the results of the HGP became available. Early studies utilised bacterial artificial chromosomes (BAC) clones for the identification of CNVs (204-207), however, with the development of SNP array technology CNV detection has become much more straightforward (24, 208, 209). The first complete CNV map of the human genome was reported in 2006 (210) and many studies have since investigated CNV and cancer risk (reviewed by Kuiper *et al*., 2010 (196)). CNV’s have been shown to have the potential to influence cancer risk by varying the gene dosage of genes involved in tumour development and progression (211), with studies focusing on the comparison between tumour and normal cells from the same individual aimed at identifying “driver genes” for the purpose of predicting prognosis and treatment (212, 213). A CNV study of over 3000 cancer specimens identified 158 CNV regions altered across the genome and found that most of the somatic CNV’s within any cancer type were common to other cancers, suggesting the existence of a combination of a limited number of functionally relevant events for cancer development (214).

Epigenetic changes are a common feature of all human cancers (215), i.e. many hyper-methylated genes have been associated with various human neoplasias (216), and can lead to genetic alteration as a result of a breakdown in key DNA repair processes like DNA mismatch repair, nucleotide excision repair and recombination repair (217). Accumulating evidence suggests that aberrant regulation or mutations of miRNAs may contribute to the pathogenesis of cancer and genomic regions containing miRNAs often are targeted for amplification, loss of heterozygosity and structural breakpoint’s in tumours (218). Recently, the complete characterisation of the microRNAome in a patient with acute myeloid leukaemia was reported, which identified novel miRNAs that were differentially expressed between the tumour and normal cells demonstrating that somatic mutations can affect gene expression (219). miRNA’s have also been show to play a role for invasion and metastasis during cancer progression (220). It has been suggested that SNPs in miRNA genes affect cancer susceptibility, response to treatment and prognosis (221). However, as miRNA SNPs are rare and minor allele frequencies low, large studies are required to confirm their relative significance.

## THE ROAD AHEAD

Next-generation sequencing, also known as massively parallel sequencing, is introducing a new era in which the poorly explored regions of the genome and their association with disease susceptibility may be revealed. In an attempt to identify the genomic landscape of cancer; targeted sequencing (for the detection of somatic mutations in cancer genomes), whole genome sequencing (matched tumour and normal genomes of a single patient) or whole transcriptome sequencing (how the somatic mutations are manifest in the genes expressed) using next-generation sequencing platforms are being utilized (reviewed by Mardis *et al.*, 2009 (189). A major challenge for all using this new technology is how to develop appropriate bioinformatics-based approaches for the data-analysis. Next-generation sequencing has already entered the clinical research arena, with targeted sequencing of 21 genes in women with severe family histories of breast or ovarian cancer proving that widespread genetic testing for personalised risk assessment can be reliably undertaken with this technology (222). New and unexpected oncogenic mechanisms have been suggested by patterns of somatic mutations discovered after sequencing 38 multiple myeloma genomes and matched normal DNA, including genes involved in protein translation, histone methylation and blood coagulation (53). Chromatin remodelling has been shown to contribute to the pathogenesis of ovarian clear cell carcinoma through exome sequencing of 8 tumours and normal cells, which identified four genes that were mutated in at least two tumours (223).

Rare genetic variants in common cancer might explain the “missing heritability” of cancer (224), however, next-generation sequencing could prove invaluable in uncovering the roles of rare variants of major effects in common disease (225). This technology will not be able to fully address the role of rare variants (including non-coding and structural variants) in cancer until it becomes a rapid cost-effective approach to analysing the entire genome (193). Nevertheless, if the cost and assay time decreases in near future, next-generation sequencing will probably be used as a general-purpose tool to characterise cancer genome for more accurate prognosis and tailored treatment of cancer patients (226).

Integrated oncology research, where a combination of genetics, epigenetics and epidemiology is used has been suggested as the key for future discoveries (227). Even sporadic tumour formation is a combination of genomic, genetic and epigenetic events and the combination of changes in gene-dosage, methylation-based silencing and polymorphisms causing reduced gene function greatly complicates the search for cancer genes (228).

## CONCLUSION

The genetic variation studies reviewed here only begin to describe the complex networks of change that seem to be involved in the development of malignancy. The pathways involved in the control of the genome are complex and poorly understood, underlining the difficulties in disease loci identification. The study of genetic variation that can contribute to complex disease is therefore a major challenge. Two main approaches exist, the candidate gene approach and the genome-wide approach and both methods have merit. The candidate gene approach can be used when possible targets exist, whereas genome-wide approach is more applicable when target of interest are yet to be identified or to identify new targets for disease development.

Linkage analysis has been used successfully in mapping genes associated with monogenic diseases (185, 229, 230), but is influenced by genetic and environmental heterogeneity and is not appropriate when identifying low penetrance tumour susceptible genes. Association studies with polymorphisms in candidate genes that are likely to affect tumour development and progression are excellent for the purpose of identifying common genetic variants that confer modest disease risk (187). But it has become very obvious to use well defined study populations when searching for genes with the candidate gene approach as it is more likely to reveal true associations between the genetic variant and disease (231). For example, a study using the candidate gene approach has identified common genetic variants in *vascular endothelial growth factor (VEGF)*, a gene critical for angiogenesis that might influence bladder cancer risk (186). Genome-wide association studies have been highly effective in exploring the role of genetic variation underlying common familial diseases (232), and have identified several susceptibility loci in common malignancies (190, 192, 199, 233).

Genetic association studies may not result in a clear understanding of the causative role of any associated genes, and are not always replicated in other studies (234). This is most likely due to the fact that the majority of the genetic variation linked to complex disease has only a modest affect on disease development, and does not adequately take into account the contribution of environmental factors to disease risk. For example, a breast cancer study examining SNPs involved in the metabolism of tamoxifen found no association with any single SNP, but when a combined SNP analysis was performed, harbouring two variant alleles in the genes *sulfotransferase family cytosolic 1A phenol-preferring member 1 (SULT1A1) and UPD glucuronosyl transferase 2 family polypeptide B15 (UGT2B15)* revealed an increased risk of recurrence and reduced survival (235). This demonstrates that it is important to consider linked polymorphisms that tend to travel through a population together, creating haplotype blocks, when searching for disease susceptibility genes (236-238).

Future cancer sequencing projects will discover the importance of mutations in cancer development and the list of cancer genes will continue to grow. It is important to incorporate epidemiological knowledge together with genetic, epigenetic and environmental studies to increase our understanding of cancer development. The appropriate application of new technologies with what we already know about cancer will lead to new screening tests and early-detection programs for high-risk relatives as well as effective population screening for common malignancies (239). There are enormous expectations about the power of next-generation sequencing and it is to be expected that significant improvements in patient outcomes will be forthcoming.

## FUNDING

This work was supported by grants from the Hunter Medical Research Institute, Cancer Institute NSW, Glady’s M. Brawn Memorial Fund through the University of Newcastle and National Health & Medical Research Council, Australia.
